# Moderate temperature shifts elicit large-scale changes in the physiology and behavior of a green microalga

**DOI:** 10.1093/plcell/koag147

**Published:** 2026-05-21

**Authors:** Barbara Bourgade, Matteo Pivato

**Affiliations:** Assistant Features Editor, The Plant Cell, American Society of Plant Biologists; Department of Organismal Biology, Uppsala University, Uppsala, Sweden; Assistant Features Editor, The Plant Cell, American Society of Plant Biologists; Department of Organelle Biology, Biotechnology and Molecular Ecophysiology, Max-Planck-Institut für Molekulare Pflanzenphysiologie (MPI-MP), Potsdam-Golm, Germany

In most natural environments, temperature fluctuations over diel and day-to-day timescales serve as a key environmental cue that shapes biological rhythms, development, and behavior across living species. The biciliate (also denoted biflagellate), unicellular green microalga *Chlamydomonas reinhardtii* naturally thrives in wet soil ecosystems, such as flooded acetate-rich rice soil, which enables mixotrophic growth through the utilization of both dissolved CO_2_ and acetate as carbon sources. Exposure of *C. reinhardtii* cells to temperatures above their reported laboratory temperature range (20 °C to 25 °C) induces pronounced regulatory and physiological changes. Moderate (35 °C) and acute (40 °C) heat stress, for example, rapidly reshapes the transcriptome and proteome, ultimately affecting DNA synthesis, photosynthesis, and carbon fixation (Zhang et al. 2022). Cold stress (5 °C) similarly triggers extensive transcriptional reprogramming (Li et al. 2020). Following a 1-h exposure to cold stress, over 3,000 genes are differentially expressed, spanning diverse biological processes, including protein synthesis, the cell cycle, and protein kinase–based phosphorylation. Together, these studies demonstrate that severe temperature fluctuations can induce coordinated system-wide responses in *C. reinhardtii*, substantially altering its regulatory landscape, metabolism, and physiology. However, even though these approaches have helped uncover key temperature-regulated pathways, they largely focus on responses to rapid and/or extreme temperature shifts. In contrast, the cellular responses to gradual and moderate changes in ambient temperature, such as those increasingly associated with climate change, remain poorly understood. Consequently, this knowledge gap raises an important question: how do algae respond to more moderate and ecologically relevant fluctuations of ambient temperature?

In recent work, Prateek Shetty and colleagues (Shetty et al. 2026) show that in mixotrophic conditions, even relatively small changes of ambient temperature trigger pronounced transcriptional, metabolic, and physiological responses in *C. reinhardtii* (see Fig. 1). Within the range of “nonstressful” temperatures tested (18 °C, 23 °C, 28 °C, and 33 °C), the higher ambient temperatures significantly affected algal-bacterial interactions, photoreceptor regulation, motility, CO_2_ consumption, and secretome composition, whereas lower ambient temperatures were associated with reduced growth and increased cell length. At the molecular level, the observed temperature-dependent transcriptional reprogramming was extensive: 5,403 genes were differentially expressed at 28 °C and 6,322 genes at 33 °C compared to 18 °C. This represents approximately one-third of all transcripts in the genome. Although a wide range of biological processes was affected, Gene Ontology enrichment analysis identified key cellular processes significantly upregulated at the higher temperatures, including extracellular matrix remodeling and flagella-related processes (Fig. 1a).

**Figure 1:**
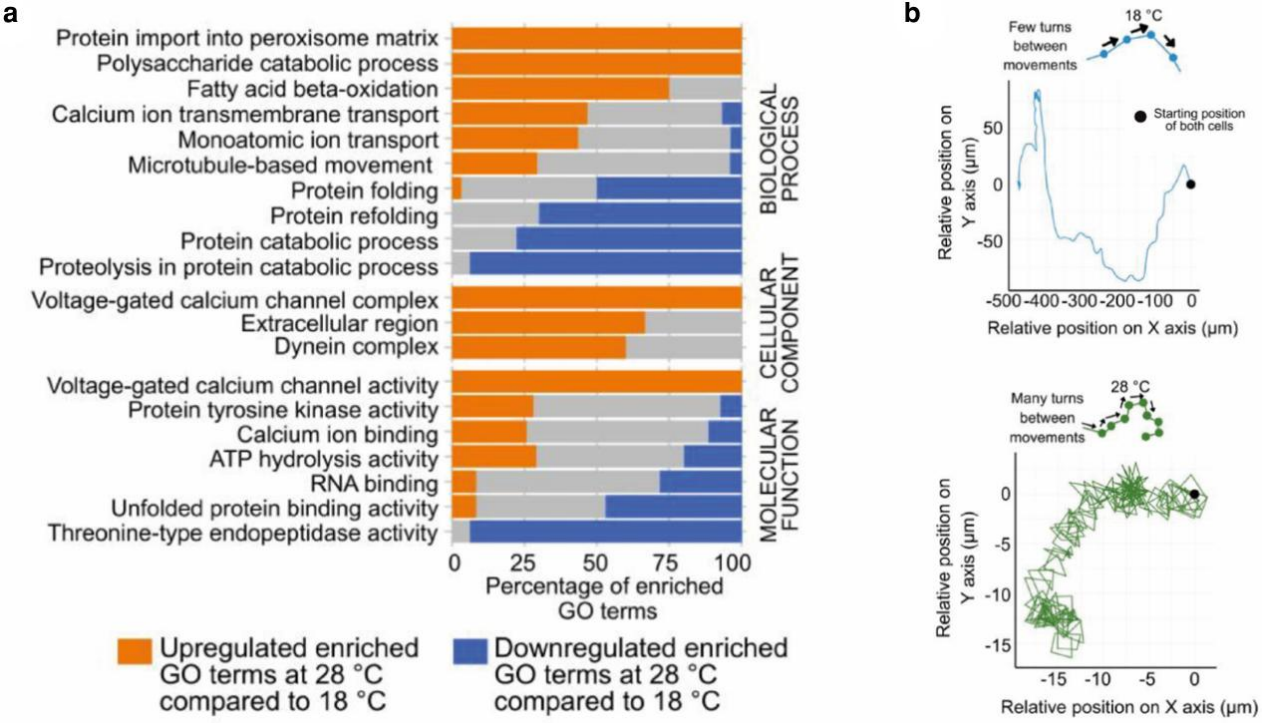
Moderate ambient temperature changes induce significant regulatory, metabolic and physiological responses in *C. reinhardtii*. **a)** Gene Ontology (GO) enrichment analysis of differentially expressed genes at 28 °C compared with 18 °C. **b)** Motility paths tracked for a representative cell cultivated either at 18 °C (upper panel) or at 28 °C (lower panel). Adapted from Shetty et al. 2026, Figures 1g; 5b and 5c.

At 28 °C the abundance of many transcripts encoding proteins of the intraflagellar transport (IFT) complexes—a crucial bidirectional transport system responsible for assembling and maintaining the flagella structure (more recently designated motile cilia)—was enhanced. Ciliary proteome analysis further confirmed this trend with 141 upregulated proteins at 28 °C, among which several IFT complex proteins were identified, suggesting enhanced IFT trafficking at elevated temperatures. This coordinated response was associated with shorter flagella at higher temperature with an average reduction of approximately 25% of their length at 28 °C compared with 18 °C. At the higher temperature, the increased abundance of proteins of the IFT trafficking machinery was reflected at the behavioral level as a significant alteration in cell motility (Fig. 1b). At 28 °C, the cells also exhibited a strong shift in their motility pattern characterized by numerous directional changes; at 18 °C the cells followed relatively straight paths, with infrequent directional changes (compare Fig. 1b upper and lower panel). Additionally, the mean swimming speed along each trajectory was significantly reduced in cells cultivated at 28 °C compared with 18 °C. While swimming path responded rapidly to short-term temperature exposure, swimming speed was partly influenced by temperature acclimation over longer exposures.

To test for temperature-dependent effects beyond motility-related processes, the authors examined the secretome. In contrast to the correlated transcriptomic and proteomic responses of ciliary components to temperature, characterization of secretome revealed opposing trends at the transcript and protein levels in response to temperature variations. While transcripts related to the extracellular matrix were downregulated, numerous secreted proteins were more abundant at 28 °C. The higher temperature elicited increases in the levels of pherophorins, which are hydroxyproline-rich glycoproteins involved in extracellular matrix (ECM) biosynthesis, and gametolysins, which are involved in cell wall degradation enabling the fusion of gametes. These findings suggest the formation of a molecular environment primed for extensive ECM modification and gametogenesis. Consistently, mating ability assays reported a higher mating efficiency at 28 °C compared with 18 °C. These data suggest that ambient temperature influences features of the sexual life cycle of *C. reinhardtii*.

Beyond regulatory and structural changes, temperature variations also triggered extensive metabolic shifts. Notably, under mixotrophic conditions, cells grown at 28 °C preferentially utilized acetate over CO_2_. In contrast, at 18 °C, cells grew primarily photoautotrophically. This finding has potential ecological implications since *C. reinhardtii* significantly contributes to O_2_ production in its natural environment: a transition toward heterotrophic metabolism upon exposure to higher temperatures could reduce O_2_ production, alter environmental levels of O_2_ and organic carbon, and thereby influence ecosystem dynamics.

In summary, Shetty and colleagues demonstrate that even moderate temperature fluctuations can lead to coordinated system-wide responses in *C. reinhardtii*, broadly affecting regulatory networks and cellular functions at both transcriptional and proteomic levels. Strikingly, key cellular processes, including motility, mating, susceptibility to bacterial attacks, and substrate consumption, are significantly modulated by small temperature variations. While climate change is severely affecting day-to-day temperature variations in many geographical areas (Liu et al. 2026), it is now clear that even relatively small temperature shifts can alter *C. reinhardtii* cell physiology and behavior in its natural habitats. In this context, this work establishes a framework for understanding how temperature changes can impact algal ecosystem dynamics.

## Recent related articles in *The Plant Cell*

- Kim et al. (2026) identified VERNALIZATION INSENSITIVE 3-LIKE 1 as a key regulator of growth retardation at low ambient temperature in Arabidopsis, where it modulates chromatin architecture at the C-REPEAT BINDING FACTOR 1 (CBF1) locus, thereby controlling CBF1-induced growth retardation.
- Li et al. (2025) showed that elevated temperature induces autophagy to selectively degrade TIMING OF CAB EXPRESSION 1 (TOC1), thereby relieving its repression of PHYTOCHROME-INTERACTING FACTOR 4 (PIF4) and promoting thermomorphogenic growth in Arabidopsis.
- Szaker et al. (2025) demonstrated that heat stress reduces transcriptional fidelity in Arabidopsis, leading to increased RNA errors that are mitigated by nonsense-mediated mRNA decay, which degrades aberrant transcripts and supports heat stress adaptation.

## Data Availability

No new data was generated for this article.
